# Hepatocyte growth factor incorporated chitosan nanoparticles augment the differentiation of stem cell into hepatocytes for the recovery of liver cirrhosis in mice

**DOI:** 10.1186/1477-3155-9-15

**Published:** 2011-04-28

**Authors:** Sivasami Pulavendran, Chellan Rose, Asit Baran Mandal

**Affiliations:** 1 Department of Biotechnology, Central Leather Research Institute, Adyar, Chennai-600020, India; 2 Chemical Laboratory, Central Leather Research Institute, Adyar, Chennai-600020, India

## Abstract

**Background:**

Short half-life and low levels of growth factors in the niche of injured microenvironment necessitates the exogenous and sustainable delivery of growth factors along with stem cells to augment the regeneration of injured tissues.

**Methods:**

Here, recombinant human hepatocyte growth factor (HGF) was incorporated into chitosan nanoparticles (CNP) by ionic gelation method and studied for its morphological and physiological characteristics. Cirrhotic mice received either hematopoietic stem cells (HSC) or mesenchymal stemcells (MSC) with or without HGF incorporated chitosan nanoparticles (HGF-CNP) and saline as control. Biochemical, histological, immunostaining and gene expression assays were carried out using serum and liver tissue samples. One way analysis of variance was used for statics application

**Results:**

Serum levels of selected liver protein and enzymes were significantly increased in the combination of MSC and HGF-CNP (MSC+HGF-CNP) treated group. Immunopositive staining for albumin (Alb) and cytokeratin 18 (CK18), and reverse transcription-polymerase chain reaction (RT-PCR) for Alb, alpha fetoprotein (AFP), CK18, cytokeratin 19 (CK19) ascertained that MSC-HGF-CNP treatment could be an effective combination to repopulate liver parenchymal cells in the liver cirrhosis. Zymogram and western blotting for matrix metalloproteinases 2 and 9 (MMP2 and MMP9) revealed that MMP2 actively involved in the fibrolysis of cirrhotic tissue. Immunostaining for alpha smooth muscle actin (αSMA) and type I collagen showed decreased expression in the MSC+HGF-CNP treatment. These results indicated that HGF-CNP enhanced the differentiation of stem cells into hepatocytes and supported the reversal of fibrolysis of extracellular matrix (ECM).

**Conclusion:**

Bone marrow stem cells were isolated, characterized and transplanted in mice model. Biodegradable biopolymeric nanoparticles were prepared with the pleotrophic protein molecule and it worked well for the differentiation of stem cells, especially mesenchymal phenotypic cells. Transplantation of bone marrow MSC in combination with HGF-CNP could be an ideal approach for the treatment of liver cirrhosis.

## Introduction

Liver cirrhosis is irreversible in many cases and leads to death if proper remedies are not taken. In recent years, numerous articles have reported the regeneration of hepatocytes or hepatocyte-like cells from stem cells [1,2]. Regeneration of hepatocytes and improvement of cirrhotic condition in mice followed by transplantation of bone marrow MSC [3,4] and cross lineage differentiation of HSC into hepatocytes [5-7] have been reported earlier. The reason for the transplantation of stem cells is to promote the regeneration of tissue specific cells and subsequent morphological and functional recovery of organs of all lineage cells [8,9]. Hence, bone marrow stem cells could be used in all ailments associated with disorders of mesodermal, ectodermal and endodermal lineage tissues. This appears to provide exciting new opportunities for stem cell therapy.

However, number of stem cells engrafted and differentiated after transplantation limit the treatment strategies. Furthermore, ambiguity continues over the contribution of which subpopulation of bone marrow stem cells actually differentiate into hepatocytes and restore the liver functions [10]. Moreover, the mechanism by which stem cells regenerate the respective parenchymal cells, heading to repair of the organs *in vivo *is yet to be completely understood. Cell fusion, in which stem cells fuse with the somatic cell in the niche, had been suggested by many authors [11] and strong evidences were reported for the transdifferentiation of stem cells [12].

Investigators attempted to improve cell therapy by a number of strategies [13] and delivery of bioactive molecules for example, growth factors, cytokines and chemokines, is one among them. Tissue repair and functional recovery after the transplantation of stem cells are augmented by the delivery of bioactive molecules that induce stem cells to differentiate into specific-lineage cells. HGF has been reported to be a potent agent for acceleration of tissue regeneration following an acute insult, as well as amelioration of tissue fibrosis and dysfunction in chronic conditions [14,15]. Though secretion of HGF after liver injury is increased, long-term secretion in the adults is questionable. Subsequently, betterment of maintenance, proliferation and differentiation of stem cells with exogenous supply of growth factors by the injured liver has been reported [7].

Despite the pleotrophic effect of HGF [16], the long term effects of exogenous HGF remain questionable because of its short half-life period. As it is also rapidly clearly by the liver *in vivo*, exogenous HGF is extremely unstable in the blood circulation with a half-life of only 3-5 min [17,18]. This makes it almost impossible to sustain a constant constantly high level of exogenous HGF in the circulation, even with repeated injections of HGF at short intervals. This necessitates the findings of the efficient alternative means to effectively deliver growth stimuli to the niche where it is needed for biological actions. Nanotechnology offers solutions for the safe and conducive transportation of therapeutic proteins to the target site [19]. Chitosan, one among the biodegradable and less antigenic natural polymers, was reported to have the potential to carry and deliver the biologically active macromolecules [20,21]. Hence, chitosan, in the form of nanoparticles, can be used to deliver HGF with less systemic dilution. Earlier our group has proven that HGF could be released from CNP and thus released HGF stimulated differentiation of MSC into hepatocyte-like cells *in vitro *[22]. Here, we demonstrated, for the first time, the ability of rhHGF incorporated CNP to differentiate MSC into hepatocytes *in vivo *followed by the decrease of severity of cirrhotic condition.

## Materials and methods

### Animal experiments

Six week old *Balbc *mice were purchased from Tamil Nadu Animal and Veterinary University, Chennai, India. Animal maintenance and handling was carried out as per the guidelines of Institutional Animal Ethics Committee. To induce liver cirrhosis, 1.0 ml/kg body weight of carbon tetrachloride (CCl4) mixed with olive oil (1:1 ratio) was injected intraperitonealy into female mice twice a week up to four weeks. Site of injection was changed on every dose to avoid necrosis of local skin and to obtain invariable results. Isolation of stem cells: MSC and HSC were isolated and characterized as per protocol [23]. Isolated cells showed typical mesenchymal and hematopoietic stem cell phenotypic characteristics. Treatment protocol: One day after the eighth injection of CCl4, MSC or sorted HSC with or without HGF-CNP or saline as a control were injected into tail vein of female mice. Either MSC or HSC of 1 × 10^6^cells was taken for injection. The amount of HGF-CNP taken for injection was adjusted such that each mouse received 100 ng of HGF. CCl4 was injected for another two weeks after cell transplantation to maintain persistent liver damage and six mice were sacrificed at predetermined time interval after post-transplantation. Liver tissue was collected after perfusing with 4% paraformaldehyde solution and preserved in formalin buffer solution for histopathological studies. For protein and total RNA isolation, liver tissue was snap-frozen in liquid nitrogen and then stored at -80°C.

### HGF incorporated nanoparticle preparation and characterization

CNP were prepared according to the protocol of Pan et.al [24]. Briefly, 0.2% chitosan (Sigma Aldrich, USA) solution was prepared in 1% glacial acetic acid (Sigma Aldrich, USA). Nanoparticles were prepared by drop-wise addition of 0.1% tripolyphosphate (TPP) (Sigma Aldrich, USA) solution into chitosan solution with or without HGF (R&D systems, USA). Turbidity was taken as an indicator for the formation of nanoparticles and the solution was subjected to centrifugation at 20,000 rpm for 20 min. The supernatant was discarded in the control and saved in the case of HGF added to quantify the amount of HGF in the supernatant by ELISA, using Human HGF Quantikine ELISA kit (R&D systems, USA) as per manufactures' instructions. All measurements were carried out in triplicate. Particle size and the morphological characteristics of the nanoparticles were examined using a high resolution transmission electron microscope (HRTEM, JEM 3010, JEOL USA, SAIF facility, IIT-Madras). Briefly, one drop of the solution containing nanoparticles was syringe placed on a carbon film (300 mesh copper grid) allowing sitting until air-dried. The sample was stained with 1% muranyl acetate solution for 5 sec at 7°C before viewing on the HRTEM.

### Evaluation of HGF encapsulation and release

HGF-CNP prepared was taken into PBS of physiological pH and kept in reciprocal shaking water bath at 37°C and 35 rpm. At predetermined time intervals, the samples were subjected to centrifugation at 20000 rpm for 20 min at 4 °C and the supernatant was replaced by fresh PBS. The amount of HGF released was quantified by ELIZA. All measurements were carried out in triplicate. The growth factor-loading efficiency of nanoparticles and their entrapment efficiency were calculated from the following equations:

### Biochemical parameters

Serum was collected to analyze alanine aminotransferase (ALT), aspartate aminotransferase (AST), and Alb. Assays were carried out at Lister Metropolis Laboratory, Chennai, India using standard automated instrumentation.

### Hydroxyproline assay

To estimate hydroxyproline content, freeze-dried liver samples were hydrolyzed in 6N HCl in sealed tubes at 110°C for 18-24 h. The hydrolyzed samples were dried over water bath and dissolved in water and then made up to a known volume. The clear supernatant obtained was used for the estimation of hydroxyproline content. The assay of hydroxyproline content was performed according to the method of Neuman and Logan [25] and the amount was expressed in μg/g wet liver tissue.

### Zymography assay

The liver protein sample (50 μg) was electrophorezed in 10% polyacrylamide gel containing 0.1% porcine skin gelatin without reducing agent. After separation, SDS was removed from the gel by two washes each 15 minutes with 1.5% Triton X-100. Subsequently, the gel was equilibrated using developing buffer (50 mM Tris [pH 7.4], 200 mM NaCl, 10 mM CaCl2, 0.02% NaN3, 1 μM ZnCl2) for 30 minutes, and incubated in the fresh developing buffer for 18-20 hr. The gel was stained with 0.25% coomasine brilliant blue (CBB) R-250 followed by destaining.

### Western blotting for MMP2 and MMP9 expression

The samples (50 μg) were resolved by 10% SDS PAGE and protein was transferred to PVDF membrane (Amersham, USA). After blocking with 5% nonfat milk, the membrane was probed with anti-mouse MMP-2 (mAB, Calbiochem, Germany). After vigorous washing with TBS, the membrane was incubated with HRP-conjugated secondary antibody (Santacruz Biotechnology, USA). Western blot was developed using diaminobenzidine substrate (Sigma Aldrich, USA) and for MMP9 detection, the membrane was probed against goat anti-mouse MMP9 (pAB, Sigma Aldrich, USA), followed by anti-goat secondary antibody for 1 hr and then, the color was developed using BCIP/NBT liquid substrate system (Sigma Aldrich, USA). The blot was photographed and semi-quantitative estimation was carried out.

### Sirius red and H&E staining

Paraffin fixed liver tissue was sectioned 5 μm size and then, the sections of liver tissue (5 μm) were stained with hematoxylin and eosin dyes for histological study. For sirius red staining, paraffin sections of 5 μm thickness were dewaxed and rehydrated and then, were stained with 0.1% sirius red (Direct Red, Sigma Aldrich, USA) in saturated solution of picric acid. Staining was photographed by light microscope (Nikon, Japan).

### Immunofluorescence assay

For immunofluorescence assay, paraffin fixed liver tissue was sectioned into 5 μm size and then, the sections were deparaffinised and hydrated. After quenching endogenous peroxidase activity with 0.3% H2O2 in methanol, blocking was carried out using bovine serum albumin (Sigma Aldrich, USA). The blocked sections were incubated overnight at 4°C against mouse Alb (pAB, abcam, USA), CK 18 and Type I collagen (mAB, Santacruz Biotechnology, USA) and α-SMA (mAB, Sigma Aldrich, USA) antibodies. The sections were incubated with FITC conjugated secondary antibodies for 15 minutes and then the slides were viewed under fluorescence microscope (Hund Wetslar, Germany). In between steps, slides were washed with PBS.

### Reverse transcription PCR analysis

Total RNA was isolated from snap frozen liver tissue using Trizol reagent (Sigma Aldrich, USA) and the ratio of absorbance values at 260 and 280 nm indicated an estimate of RNA purity. RT-PCR was performed using one-step RT-PCR kit (Qiagen K.K., Tokyo, Japan) with the following primers: CK18 S: A: 5'-TGGTACTCTCCTCAA TCTGCTG-3', A:5'-CTCTGGATTGACTGTGGAAGTG-3' (148 bp), CK19 S:5'-CATGGTTCTTCTTCAGGTAGGC-3', A S:5'-GCTGCAGATGACTTCAGAACC-3' (291 bp), Alb S:5'-TCAACGTCAGAGCAGAGAAGC-3', A: 5'-AGACTGCCTTGTGTGGAAGACT-3', (145), AFP S: 5'-GTGAAACAGACTTCCTGGTCCT -3', A: 5'-GCC CACAGACCATGAAACAAG-3'(bp148). RT-PCR was used to evaluate chimerism in mouse liver tissue after sex mismatched stem cell transplantation. Male derived MSC and HSC were transplanted into female mice. Primers for *sry *gene specific for mouse testis were selected from the previous study. Forward and reverse primers were as follows: F5'AGAGATCAGCAAGCAGCTGG 3', R5' TCTTGCCTGTATGTGATGGC 3' (bp248).PCR reactions were performed according to manufacturer's instruction with each cycle in a Eppendorf Thermal Cycler (Takara, Tokyo, Japan) using appropriate cycle profile. After the reaction, aliquots of the product were run on 1% agarose gel, stained with ethidium bromide. The amount of amplified product was quantified for each sample using a computing densitometer (Gel Doc EQ Gel documentation System; Biorad Laboratories, Hercules, CA) and software (Quantity One). The final amount of PCR product was expressed as the ratio of the respective gene amplified to that of the βactin gene, to account for any differences in beginning amounts of RNA.

### Data Analysis

Experimental results were expressed as mean±S.D. Analysis of variance was performed by one way analysis of variance procedures (SSPS 9.0 for Windows). Significant differences between means were determined by Dunnett's post hoc test.P < 0.05 implies statistical significances. For histopathological assays, the sections were taken from multiple samples at various locations. The best out of these figures is given for representation in each group.

## Results

### Physicochemical characteristics of HGF incorporated nanoparticles

**Figure 1:**
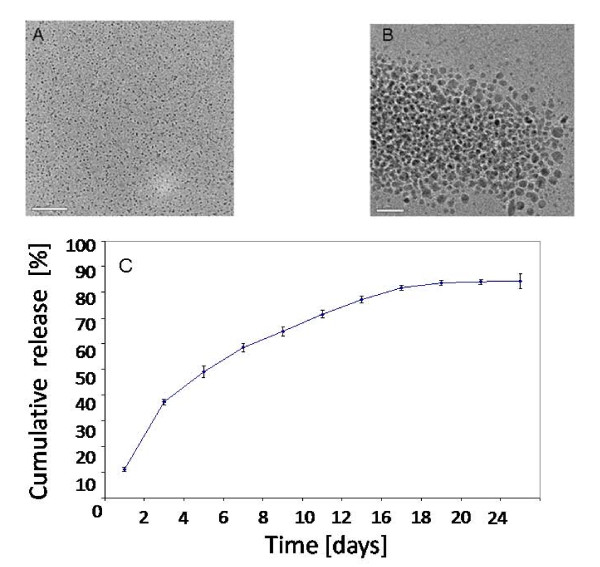
**Chracterization of nanoparticles**: High resolution TEM of (A) chitosan nanoparticles (B) HGF incorporated nanoparticles. Particles are flat and round shape (C). Cumulative release pattern of HGF from CNP. Sustainable release of growth factor could be seen from the release curve. Values are presented as mean ± S.D for triplicate experiment.

Morphological characteristics of CNP and the release pattern of HGF from CNP were studied. The loading efficiency of nanoparticles and entrapment efficiencies of HGF were 5 ng HGF/mg of nanoparticles and 85% respectively. This was achieved with chitosan to TPP ratio at 2:1. At lower ratio, more protein could be incorporated since more positive charge from chitosan will be available. Care was taken not to form crystal-like particles during the preparation. Plain and HGF loaded nanoparticles observed by HRTEM (Figure 1 A and 1B) appeared spherical in shape with a particle size range of 50-100 nm. The *in vitro *release profile of HGF from the nanoparticles in PBS at the predetermined time was expressed as percentage of HGF released with respect to the total amount of HGF encapsulated (Figure 1C). HGF release observed after 24 and 35 days was 82 and 85% respectively, indicating the monophasic release pattern of nanoparticles over a period of at least 25 days. This indicates that physical adsorption of the growth factor on the nanoparticles is unlikely, which is an essential characteristic feature for the nanoparticles required for sustained release.

### MSC-HGF-CNP improved liver morphology, function and hepatocytes proliferation

The liver samples of control and HSC treated groups appeared pale and shrunken and the samples treated with MSC/+HGF-CNP were reddish brown and normal (data not shown). The liver function tests of each group after fourth week of transplantation was assessed by analyzing the serum levels of Alb, ALT and AST and the results are presented in Table 1. Elevated levels of Alb and aminotransferase enzymes were found in control, HSC and HSC+HGF-CNP treated groups. Significant increase of Alb (2.5 ± 0.03d, dP < 0.001) and decreased levels of ALT (95 ± 5d,dP < 0.001) and AST (510 ± 15c, cP < 0.05) were found in the MSC/+HGF-CNP treated group. These findings indicated that MSC/+HGF-CNP treatment induced the secretion of these liver specific proteins.

**Figure 2:**
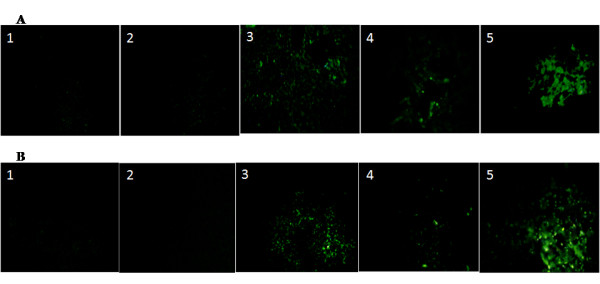
**Immunofluoresent expression**: After 4^th ^week of post-transplantation, the liver was perfused with 4% paraformaldehyde solution and stored in 10% buffered formalin solution. After dewaxing and dehydrating, the sections were incubated overnight with anti-mouse Alb (A) and CK18 (B) antibodies and the expression of these proteins was viewed by fluorescent microscopy using secondary antibody tagged with FITC. Both Alb and CK18 were highly expressed in MSC and MSC+HGF-CNP treated groups but not in control, HSC and HSC+HGF-CNP treated groups. It displayed the differentiation of MSC and augmentation of differentiation by HGF-CNP (Control magnification 100 ×).

**Figure 3:**
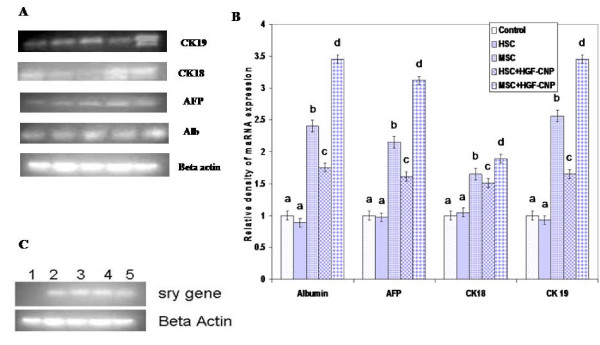
**Gene expression of liver proteins**: (A) Total RNA was isolated using Trizol agent and after quality verification, PCR was carried out using PCR kit and the products were run on 1% agarose gel to analyse the expression of genes of Alb, AFP, CK18 and CK19. Lane:1 Control, Lane:2 HSC, Lane:3 MSC, Lane:4 HSC+HGF-CNP, Lane:5 MSC+HGF-CNP. All these genes were highly expressed in MSC treated groups compared to control and HSC treated groups. (B) Semiquantification of liver specific proteins - mRNAs Alb, AFP, CK18 and CK19 of control and treated groups were significantly expressed in MSC and MSC+HGF-CNP treatment. In control and HSC treated groups, expressions were not significant. (C)RT-PCR expression of *sry *gene (Male Y chromosome specific gene). *Sry *gene is expressed in all treated groups, showing the presence of chimerism. in all groups.

To verify hepatic differentiation of transplanted stem cells and subsequent recovery of liver cirrhosis, the expression of liver markers such as Alb and CK18 was examined in control and treated groups by immunostaining. Alb and CK18 proteins increasingly expressed in the MSC/+HGF-CNP treated group compared to other groups (Figure 2A and 2B) despite continuous injection of CCl4 after post-transplantation. On the other hand, the expression of these markers was less in the control and HSC/+ HGF-CNP treated groups. This observation is in good correlation with the results of biochemical parameters. The results of both biochemical and immunohistochemical studies were further confirmed by the expression of mRNAs of Alb, CK18, AFP and CK19 proteins (Figure 3A). In semiquantitative analysis of gene expression, all of these genes expressed significantly (P < 0.05). HSC, even in the presence of HGF-CNP, did not show significant increase in the expression of these proteins and their levels were observed to be less than in MSC treated groups. Analysis of chimerism after cell transplantation is important for assessing the graft by the presence of donor cells. It is usually detected by the expression of specific gene by donor cells. *Sry *gene from donor male cells was detected by RT-PCR. *Sry *gene was expressed in all treatment groups; however, the control group did not show *sry *gene expression (Figure 3C).

In control and HSC treated groups, these genes were not expressed significantly. From this, it was confirmed that the transplanted MSC were able to differentiate into liver parenchymal cells, wherein HGF-CNP enhanced the process of differentiation. Wang et al (2003) showed that hepatic differentiation of HSC could be enhanced by intravenous injection of soluble HGF. None of the earlier reports explained the receptor mechanism or pathways with which HGF facilitates the differentiation of HSC after transplantation. ***S****ry *gene expressed in all transplantation experiments except control (Figure 3C). RT-PCR result for engraftment shows that chimerism in HSC treated mice is possible but the exact place where the transplanted cells is oriented is still questionable and whether undifferentiated HSC or differentiated parenchymal cell or differentiated nonparenchymal cell contribute for the chimerism is also to be understood.

### Histology and collagen content

**Figure 4:**
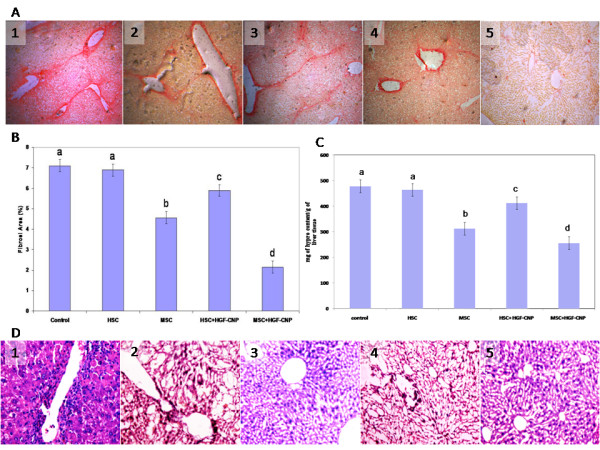
**Histological appearance of the liver**: (A) Expression of collagen was viewed by sirius red staining: Collagen bridge was uniform in control and HSC treated groups but not in MSC and MSC+HGF-CNP treated groups (original magnification × 100). (B) Quantitative analysis of fibrosis is shown in histographic representation. The degree of fibrosis was expressed as the percentage of the total area measured. The fibrosis areas were significantly low in MSC and MSC+HGF-CNP treated groups (P < 0.05). However there were no statistical differences in liver fibrosis between control and HSC treated groups. (C) Hydroxyproline content was estimated by Neuman and Logan method. Hydroxyproline content did not vary significantly between control and HSC treated groups and but was significantly low in MSC and MSC+HGF+CNP groups (P < 0.05). (D) Formalin fixed liver tissues were sectioned into 5 μm size and stained with H&E dyes. Infiltration of inflammatory and necrotic cells was ubiquitous in control and HSC treated groups however, MSC treated groups did not show inflammatory cells (original magnification ×100) Control (1); HSC (2); MSC (3); HSC+HGF-CNP (4); MSC+HGF-CNP (5).

Fibrotic conditions were assessed after fourth week of transplantation using sirius red staining and hyproxyproline content of the cirrhotic tissue. The representative images for fibrosis are shown in Figure 4A. Distribution of fibrosis was extensively reduced in MSC/+HGF-CNP group followed by MSC alone compared to control, HSC or HSC+HGF-CNP treated groups. Quantification of fibrosis by image analysis (Figure 4B) clearly indicated that the level of fibrosis has been significantly reduced in MSC, 4.56 ± 0.29 (P < 0.05) and MSC+HGF-CNP, 2.15 ± 0.14 (P < 0.01) treated groups but not in HSC (6.89 ± 0.24) or HSC+HGF-CNP (5.89 ± 0.18) groups compared to control. The hyproxyproline content (Figure 4C) in HSC or HSC+HGF-CNP treated groups did not show significant difference from the control group; however in the case of MSC or MSC+HGF-CNP treated groups, the hyproxyproline content was significantly low compared to the control (P < 0.01). These results provided direct evidence for an antifibrotic effect of MSC and MSC + HGF-CNP combination. Figure 4D shows the histology of liver tissue of control and treated groups. The microscopical view of the H&E stained specimens revealed the appearance of disrupted tissue architecture with large fibrous septa and infiltration of inflammatory and necrotic cells in the liver sections of control, HSC or HSC+HGF-CNP treated groups but not in MSC and MSC+HGF-CNP treated groups.

### MSC suppress the activation of hepatic stellate cells

**Figure 5:**
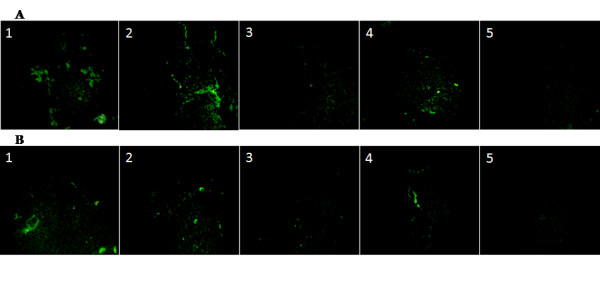
**Immunofluoresent expression of ECM proteins**: After 4th week, livers were harvested after perfusing with 4% paraformaldehyde solution and stored in 10% buffered formalin solution. For immunofluoresence assay, the specimens after dewaxing and dehydrating were blocked for nonspecific protein using BSA. Then, the sections were incubated overnight with anti-mouse α-SMA antibody (A): Control (A1), HSC (A2), MSC (A3), HSC+HGF-CNP (A4) and MSC+HGF-CNP (A5); and anti-mouse type I collagen antibody (B): Control (B1), HSC (B2), MSC (B3), HSC+HGF-CNP (B4) and MSC+HGF-CNP (B5). The expression of these proteins was viewed by fluorescent microscopy using secondary antibody tagged with FITC. Both α-SMA and type I collagen expressed high in control, HSC and HSC+HGF-CNP treated groups than MSC and MSC-HGF-CNP treated groups. MSC treatment suppressed the myofibroblasts and consequently made type I collagen disappeared (original magnification ×100).

Inflammatory cascade activates the quiescent hepatic stellate cells into myofibroblasts which was confirmed by the immunostaining of α-SMA (an indicator of activated myofibroblasts). Secretion of type I collagen by myofibroblasts that leads to fibrosis was analyzed by immunofluorescence. The results of imunofluorescence study for distribution of α-SMA positive cells around the sinusoid portion (Figure 5A) confirmed the histological observation (Figure 4D) in respect of the presence of large number of nonparenchymal cells with consistant morphology of activated myofibroblast-like cells clustered around the fibrotic septae at the end of fourth week in control, HSC or HSC+HGF-CNP treated groups. Increased expression of α-SMA in the periportal region of liver of control, or HSC+HGF-CNP treated groups and the reduced expression of this protein in MSC and MSC+HGF-CNP groups were also noticed. Decreased expression of type I collagen, due to reduced myofibroblast activity, in MSC or MSC+HGF-CNP compared to control and other treated groups is shown in Figure 5B.

### MMPs activity and expression

**Figure 6:**
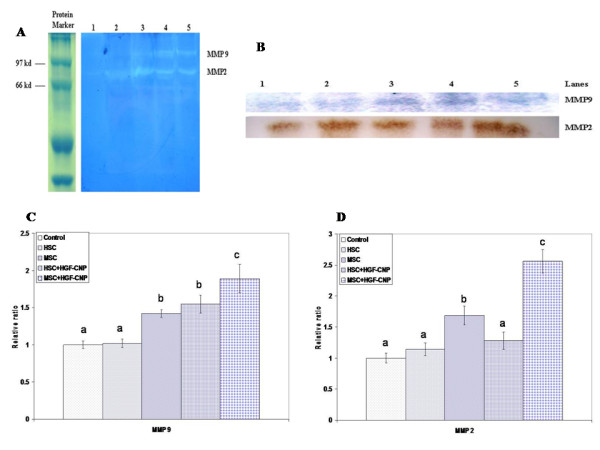
**Expression of MMPs**: (A) The activity of MMPs was analysed by zymography. The liver homogenate was resolved in 10% SDS-PAGE containing 0.1% gelatin without reducing agent. The gel was then stained and washed. MMPs were well expressed in MSC and MSC+HGF-CNP groups; particularly, MMP2 was increasingly expressed in these groups compared to control, HSC and HSC+HGF-CNP treated groups. This indicated the activation of MMPs by MSC. (B) Expression of MMPs was analyzed by western blotting. Electrophoretically resolved proteins were transferred and then immunostained against mouse MMP2 and MMP9. Control group 1, HSC 2, MSC 3, HSC+HGF-CNP 4, MSC+HGF-CNP 5 treated mice showed increased expression of MMPs, particularly MMP2. Semiquantitative analysis of MMP2 and MMP9 was carried out and significant level of expression of MMP2 and MMP9 was found in MSC and MSC+HGF-CNP treated groups (P < 0.05) compared to control, HSC and HSC+HGF-CNP treated groups. Significant level of expression of MMP2 appeared compared to MMP9. It explored the reason for the decrease of ECM.

The fibrolytic activity of MMPs and their expression are depicted in Figure 6A and 6B respectively. The zymography assay revealed a band of gelatin degradation at 68 kDa, representing active MMP-2 and another band at 97 kDA representing MMP9 (Figure 6A). The MSC groups, particularly MSC+HGF-CNP, showed increased expression of MMPs, more especially MMP2. The overall data on the quantitative analysis of MMP9 (Figure 6C) and MMP2 (Figure 6D) indicated that MMP9 was relatively less expressed and active compared to MMP2.

## Discussion

The present study described the effect of HGF-CNP on the *in vivo *hepatic differentiation of stem cells. Earlier, increased expression of Alb was reported after exogenous injection of rhHGF in mice [7]; however, *in vivo *availability of HGF was not considered in the study since its half-life period is very short [17,18]. To maintain adequate level of serum HGF and overwhelming its short half-life period, repeated injection of HGF [26] and/or its gene [27] was suggested. In this study, we have incorporated HGF into CNP by ionic gelation method. Such particles can carry the therapeutic proteins to the targeted site without degradation and can sustain in the circulatory system. The protonated amino groups of chitosan as well as HGF in the acidic medium electrostatically combined with anions of TPP to form cross-linkage and this procedure ensured the systemic incorporation of growth factor into the nanoparticles instead of adsorption. Nanoparticles having the size range of 50-200 nm could be used effectively for the biological application by injecting them intravenously because they are capable of reaching multiorgans for therapeutic application [28]. CNP with 50-200 nm size prepared in this study (Figure 1) showed sustainable *in vitro *release up to 24 days as against the earlier report where the release was observed only for 8 days [29]. The cumulative release of 82% of HGF after 24 days observed in our study indicated an extended time course for sustainable release ruling out the possibility of either biphasic or burst release. This level of controlled release (4 ng per day) could be sufficient enough to induce differentiation of stem cell as has been observed by Hasuike et al. [30]. Another important feature of this study was the effective use of HGF to a level of, as low as, 1.2 μg HGF/mg CNP/kg body weight, against 250 μg/Kg body weight [31] and 300 μg/Kg body weight [26] reported earlier.

Less engraftment of transplanted cells necessitated the findings of effective strategies which can differentiate and expand the transplanted cells. The process of migration of MSC to the target site was reported to be guided and accelerated in the presence of HGF [32]. the histological findings of our present study suggests that the exogenous HGF prevent the hepatocytes from necrosis and accelerated regeneration [27] as was observed in our histological results. Results of these previous studies brought forth the idea of delivery of HGF through carriers, especially nano-carrier, to aid the targeted as well as sustainable delivery. In this study we report that HGF released from HGF-CNP could also accelerate the migration of MSC to injured liver and also facilitated its hepatic differentiation, as only these cells have c-met receptor for HGF. This was supported from the results of increased expression of liver specific proteins and their genes (Figure 2 and 3). From these results, it was confirmed that the transplanted MSC were able to differentiate into liver parenchymal cells, wherein HGF-CNP helped to enhance the differentiation. Wang et al. [7] showed that hepatic differentiation of HSC could be enhanced by intravenous injection of soluble HGF. None of the earlier reports explained the receptor mechanism or pathways with which HGF facilitates the differentiation of HGF after transplantation.

Differentiation of homed stem cells at the target site was monitored by the expression of *sry *gene after transplantation of stem cells in sex mismatched mice. The expression of *sry *gene confirmed the engraftment of both HSC and MSC in the recipient's liver. RT-PCR result for engraftment showed that chimerism in HSC treated mice is possible but the exact place where the transplanted cells is oriented is still questionable and which undifferentiated HSC or differentiated parenchymal cells or differentiated nonparenchymal cell contributed for the chimerism is also to be understood. Higher expression of *sry *gene observed in the MSC+HGF-CNP treated group ascertained the migration followed by engraftment for the effective repopulation of tissue-specific hepatocytes. The HGF-incorporated chitosan nanoparticles can presumably work for the differentiation process in two ways: injected HGF-incorporated nanoparticles may release the growth factor in the circulation during the controlled enzymatic degradation of biopolymers and thus released HGF may enhance the differentiation; secondly, the liver being the homing organ for any foreign particles, the CNP upon reaching the liver is degraded to release the growth factor which can induce the differentiation.

Amelioration of fibrosis and its grounds must be suppressed or stopped to prevent progression of fibrosis. Hepatic injury activates the secretion of cytokines of inflammatory cascade from the multiple inflammatory as well as parenchymal cells, which involve the healing process. Suppression of inflammatory cytokines could reduce the activation of hepatic stellate cells. Inhibition of the proliferation of T cells thereby modulating the pro-inflammatory cytokines such as TNF-α and IL1βby MSC was reported [33-35]. Less invasions of inflammatory cells in the MSC and MSC-+HGF-CNP treated groups connect with the anti-inflammatory action of MSC. Moreover, the direct involvement of MSC in immunomodulation of hepatic stellate cells has also been explored recently [36]. Lower level of α-SMA positive cells in MSC treated group (Figure 5) is attributed to the anti-inflammatory activity of MSC that secrete compounds which would have reversed myofibroblasts through paracrine mechanisms.

Most of the previous studies involving the transplantation of stem cells for therapeutic purpose, concentrated on either functional recovery of liver from metabolic disease in the knockout model [37] or the fibrolysis of ECM content [38]. For better understanding we have compared the contribution of either HSC or MSC in the process of reversal of cirrhosis in the liver. A significant difference was observed in collagen content between MSC and HSC treated groups or control (Figure 4). The disappearance of collagen content in the cirrhotic liver of MSC groups was apparently due to the lysis of fibrotic tissue which was accomplished by MMP2 activity. MMPs more particularly the MMP2 that promote the degradation of ECM in liver cirrhosis [39] should have been secreted by MSC, which in the presence of HGF exhibited increased MMP2 activity, as observed by enhanced fibrolysis and/or prevention of collagen synthesis. This would have facilitated the assembling and orientation of stem cells in the hepatic nodules where they can differentiate into functional hepatocytes. Though HSC can differentiate and recover the liver functions to some extent, they obviously failed to degrade the ECM. The expression of MMPs by HSC either in *in vitro or in vivo *studies has not been reported so far. But it was reported that transplanted HSC activate T lymphocytes leading to inflammatory complications and posing health risk in hematopoietic stem cell therapy [40]. Moreover, in the cirrhotic liver, therapeutic strategies must rely on achieving repopulation of liver parenchymal cells and increased ECM degradation [41]. The potential of MSC for differentiation, immune-suppression and the secretion of matrix degradation molecules suggested that MSC based cell therapy could be used successfully for the treatment of liver inflammation and cirrhosis. This model could also be extended to the reversal of other fibrotic condition. Our further study will be extended in the direction of *in vivo *kinetics, distribution and stability of HGF-CNP in the blood circulation. Whether HGF released from the CNP causes the stem cell differentiation or apoptosis of myofibroflasts or both of these functions must be studied in detail with the appropriate controls such as HGF, CNP and HGF-CNP alone to derive the concept to meaningful clinical applications.

## Conclusions

Enhancement of regenerative effects of stem cells for the treatment of tissue injuries and genetic developmental diseases could be carried out with the multiple strategies such as gene therapy, delivery of therapeutic proteins etc. Development of biodegradable delivery devisees for the regenerative medicine is the urgent need to compensate/enhance the slow differentiation of stem cells. HGF incorporated CNP prepared in this investigation showed appreciable morphological and kinetic properties and it enhanced the differentiation of stem cells *in vivo*, especially mesenchymal phenotypic cells. Transplantation of bone marrow MSC in combination with HGF-CNP was seen as an ideal approach for the treatment of liver cirrhosis. This study will be extended in the direction of *in vivo *kinetics, distribution and stability of HGF-CNP to focus further on the localized delivery with the receptor mechanism.

## Competing interests

The authors declare that they have no competing interests.

## Authors' contributions

SP has carried out the preparation of HGF-incorporated chitosan nanoparticle and biochemical, histological, immunostaining and gene expression assays described in the manuscript, statistical data analysis and has drafted the manuscript. CR has provided the interpretation of data of the entire manuscript and has finalized the manuscript contents with critical revisions. ABM has suggested valuable inputs with interpretation of statistical data and has duly approved the manuscript for submission to the Journal.
